# Subthalamic Deep Brain Stimulation Lead Asymmetry Impacts the Parkinsonian Gait Disorder

**DOI:** 10.3389/fnhum.2022.788200

**Published:** 2022-03-28

**Authors:** Frederik P. Schott, Alessandro Gulberti, Hans O. Pinnschmidt, Christian Gerloff, Christian K. E. Moll, Miriam Schaper, Johannes A. Koeppen, Wolfgang Hamel, Monika Pötter-Nerger

**Affiliations:** ^1^Department of Neurology, University Medical Center Hamburg-Eppendorf, Hamburg, Germany; ^2^Department of Neurophysiology and Pathophysiology, University Medical Center Hamburg-Eppendorf, Hamburg, Germany; ^3^Department of Medical Biometry and Epidemiology, University Medical Center Hamburg-Eppendorf, Hamburg, Germany; ^4^Department of Neurosurgery, University Medical Center Hamburg-Eppendorf, Hamburg, Germany

**Keywords:** deep brain stimulation, subthalamic nucleus, Parkinson’s disease, balance, gait disorder, freezing of gait, electrode localization, lead asymmetry

## Abstract

**Background:**

The preferable position of Deep Brain Stimulation (DBS) electrodes is proposed to be located in the dorsolateral subthalamic nucleus (STN) to improve general motor performance. The optimal DBS electrode localization for the post-operative improvement of balance and gait is unknown.

**Methods:**

In this single-center, retrospective analyses, 66 Parkinson’s disease (PD) patients (24 female, age 63 ± 7 years) were assessed pre- and post-operatively (8.45 ± 4.2 months after surgery) by using MDS-UPDRS, freezing of gait (FoG) score, Giladi’s gait and falls questionnaire and Berg balance scale. The clinical outcome was related to the DBS electrode coordinates in x, y, z plane as revealed by image-based reconstruction (SureTune™). Binomial generalized linear mixed models with fixed-effect variables electrode asymmetry, parkinsonian subtype, medication, age class and clinical DBS induced changes were analyzed.

**Results:**

Subthalamic nucleus-deep brain stimulation improved all motor, balance and FoG scores in MED OFF condition, however there were heterogeneous results in MED ON condition. DBS electrode reconstructed coordinates impacted the responsiveness of axial symptoms. FoG and balance responders showed slightly more medially located STN electrode coordinates and less medio-lateral asymmetry of the electrode reconstructed coordinates across hemispheres compared to non-responders.

**Conclusion:**

Deep brain stimulation electrode reconstructed coordinates, particularly electrode asymmetry on the medio-lateral axis affected the post-operative responsiveness of balance and FoG symptoms in PD patients.

## Introduction

The parkinsonian (PD) gait disorder with freezing of gait (FoG) and balance disturbance is a common and incapacitating symptom with high impact on quality of life (Moore et al., 2007; Okuma, 2014). The treatment of the PD gait disorder remains quite challenging (Nonnekes et al., 2015). Beside dopaminergic medication, deep brain stimulation (DBS) of the subthalamic nucleus (STN) represents one therapeutical option, however the effects of STN-DBS on balance and gait are heterogeneous (Potter-Nerger and Volkmann, 2013). STN-DBS might have a positive impact on balance (Sato et al., 2019; Li et al., 2020) and FoG (Schlenstedt et al., 2017; Barbe et al., 2020), however despite stable improvements of global outcome scores after bilateral STN-DBS, there are also reports of post-operative worsening of gait (van Nuenen et al., 2008), increased risk of falls (Hausdorff et al., 2009) or persistent levodopa-resistant freezing of gait (Stolze et al., 2001). Long-term observations (>5 years) revealed a decrease of DBS effects on axial symptoms (Krack et al., 2003; Mei et al., 2020). Different factors might contribute to these heterogeneous gait effects of STN-DBS, as disease progression, age (Russmann et al., 2004) or the pre-operative levodopa-response (Bakker et al., 2004; Schlenstedt et al., 2017). One factor impacting gait outcome might be the exact lead localization of the STN electrode (Johnsen et al., 2010).

There are reports of differential effects on global motor outcome in terms of DBS electrode position (Johnsen, 2011). Systematic investigation of the different electrode contacts in the vertical axis in relation to anatomically and electrophysiologically defined STN boundaries revealed, that contacts located at the dorsolateral border of the STN had the best effect on contralateral appendicular motor symptoms (Hamel et al., 2003; Herzog et al., 2004). Further detailed analyses of axial MRI planes revealed that positioning of the lead in the anterolateral dorsal STN predicted the best general motor outcome (Wodarg et al., 2012). In terms of gait and balance improvement, the optimal electrode position within the STN and the relative position of DBS electrodes to each other across both hemispheres is less clear. One early study in a small PD cohort investigated the correlation of the position of the DBS electrode and outcome on objective measures of gait (Johnsen et al., 2010). Stimulation of contacts located in the dorsal half of the STN was more effective in improving step velocity and step length of the contralateral leg compared to ventral stimulation being in line with general motor symptom improvement (Johnsen et al., 2010).

The aim of the current, monocentric, retrospective analyses was to assess the effect of stereotactic DBS electrode localization within and across hemispheres on the post-operative outcome of the parkinsonian gait disorder and dissect these effects from other potential influencing factors as age, pre-operative symptom severity and levodopa-responsiveness of the parkinsonian gait disorder.

## Materials and Methods

### Patient Characteristics

Sixty six patients (24 female, age 63 ± 7 years) suffering from advanced idiopathic PD (disease duration 10.41 ± 3.65 years; Hoehn and Yahr stage: 2.6 ± 0.81) were included into the retrospective analysis from clinical routine data. Inclusion criteria were 1. PD in Hoehn and Yahr 2–5, 2. Implantation of Medtronic, Boston Scientific or Abbott DBS systems, 3. Stable post-operative condition (>3 months, <1 year) 4. PD patients were not stimulated with a bipolar configuration.

Parkinsonian patients were screened and selected for DBS surgery in accordance to common guidelines of DBS surgery [CAPSIT protocol (Defer et al., 1999)]. Of all 66 patients, 17 patients were classified as tremor-dominant PD subtypes, 41 patients as akinetic-rigid subtypes and eight patients as equivalent subtypes.

Further clinical and demographic characteristics of PD patients are reported in Supplementary Table.

### Implantation of the Permanent Deep Brain Stimulation Electrodes

Deep brain stimulation electrode placement was guided by intraoperative microelectrode recording (MER) and test stimulation. Up to five parallel tracks were used to map the subthalamic region with tungsten electrodes (NeuroProbe electrodes, Alpha Omega Inc., Nazareth, Israel). The subthalamic sensorimotor region was identified by cell responses to passive and active movements and a high prevalence of oscillating neuronal activities in the beta-frequency range (13–30 Hz). Permanent macroelectrodes were inserted in the best MER track with longest ventro-dorsal electrophysiological recording of STN activity and optimal clinical test stimulation effects.

### Clinical Scores

Clinical assessments were performed pre-operatively after overnight withdrawal of medication (MED OFF) and after application of suprathreshold dosage of soluble dopaminergic medication (MED ON) to explore short-term dopaminergic effects. Post-operatively (8.45 ± 4.2 months after surgery), PD patients were assessed with DBS switched on (STIM ON) in MED OFF and MED ON. The following clinical scores were routinely applied:

1. The MDS-UPDRS part III score was used to assess general motor performance. The lateralized subitems (items 3.3–3.8, 3.15–3.18) were summarized. We calculated asymmetry scores from the lateralized items from the worst and best clinical side [(lateralized MDS-UPDRS worst side-lateralized MDS-UPDRS best side)/worst side] with asymmetry scores of 0 indicating perfect symmetry and 1 revealing most severe asymmetry.
2. The Ziegler’s freezing of gait assessment course score (FoG score) (Ziegler et al., 2010) was used as short-interval rater-based scale to quantify festination and FoG.
3. The Giladi’s gait and falls questionnaire (GFQ) (Giladi et al., 2009) was applied as a 16 items questionnaire reflecting the patient’s subjective perspective on falls and FoG pattern.
4. The short version of the Berg balance scale (Chou et al., 2006) was assessed as a seven item rater-based balance score.

### Localization of Electrodes and Active Contacts

**FIGURE 1:**
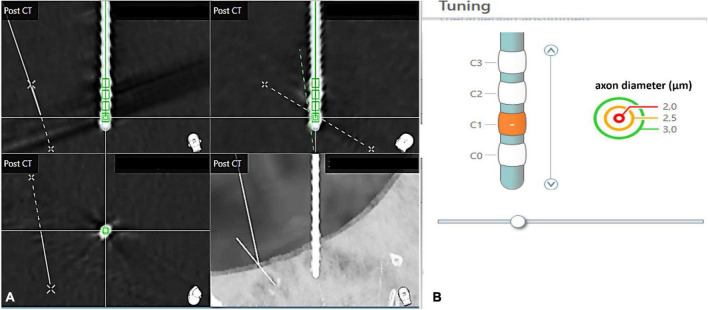
Methodological approach of electrode localization within the SureTune™ software. After definition of the AC-PC line in the axial and sagittal plane in the merged CT and MRI images, determination of spatial parameters of electrode localization was performed **(A)**. Definition of active contact and modeling of volume of tissue activated by using corresponding stimulation parameters **(B)**.

Magnetic resonance imaging (MRI) scans (Siemens Skyra, 3 Tesla, 0.94–1.6 mm slice thickness, TR 2100, TE 2.5, FA 9.0) were obtained from all PD patients pre-operatively. MRIs were fused with post-operative CT scans (Siemens Somatom Definition AS, 1 mm slice thickness, RD 200, MA 154, KV 120, FOV 200 mm × 200 mm). The reconstruction of the stereotactic electrode position in the x, y, z plane was performed by using SureTune™ software version 3.0.3.0 licensed by the Medtronic Company (Figure 1). The anterior (AC) and posterior commissure (PC) as well as the inter-hemispheric plane (IH) were defined in pre-operative, T1- weighted MRI (Hamel et al., 2003) and represented the axes of the three-dimensional coordinate system in which the electrodes coordinates were determined. The electrode reconstruction was performed by one main analyst and controlled by two experienced neurosurgeons of the local stereotactic neurosurgical department, who are doing routinely the DBS surgeries.

Magnetic resonance imaging (MRI) data were fused with pre- and post-operative CT scans in order to co-register the various images to the same reference. Individual contacts of DBS leads were displayed by the software after the DBS lead type had been specified (Boston Scientific, Abbott and Medtronic). The investigator performing reconstruction of DBS leads was blinded to clinical outcomes. The stereotactic x-, y-, and z-coordinates of the stimulated contacts were calculated. The stimulation parameters (amplitude, pulse width, pulse frequency) were used to calculate the volume of tissue activated (VTA).

Changes in the clinical scores between the pre- and post-operative state were correlated with the DBS localization on the medio-lateral (x-axis), anterior-posterior (y-axis), dorso-ventral line (z-axis) used by the neurosurgeons to define the target area of the STN referring to the midcommissural point. Electrode asymmetry of the two electrodes across hemispheres were analyzed by measuring the absolute distance to the midcommissural point in the right and left hemisphere respectively, and calculating the difference of x right-x left, this was repeated for the y and z reconstructed coordinates. A difference of 0 indicated optimal symmetry of the right and left electrode relative to the midcommissural point.

### Statistics

In a first step, descriptive scores were reported as means and standard deviations of the mean (SD). Clinical scores pre- and post-operatively were compared by paired *t*-tests after testing for normal distribution. The VTA by DBS electrodes and levodopa equivalent daily dose (LEDD) were correlated with post-operative changes of the different motor scores by linear regression analysis and non-parametric Spearman tests.

In a second step, patients were subdivided into two groups depending on the responsiveness to the STN-DBS treatment. If the difference between the post-operative scores minus the pre-operative scores was negative in case of FoG score, Giladi’s GFQ and MDS-UPDRS-III, and positive in case of the Berg balance scale, indicating a post-operative improvement of the scores, the patients were assigned to the responder group, otherwise to the non-responder group. The responsiveness to therapy was analyzed using a generalized linear mixed model approach with a logit-link function assuming binomially distributed data (SPSS routine generalized linear mixed models; IBM SPSS Statistics for Mac, version 25.0.0.2, SPSS Inc., Chicago, IL, United States). Prior to analysis, all continuous variables with a positively skewed distribution were log for base 2 transformed to achieve normal distribution [log2x = ln(x)/ln(2)]; negatively skewed distributions were first reverse-score transformed before the log for base 2 transformation (Field, 2009). The asymmetry index of the x, y, z stereotactic coordinates (the absolute values of the difference between x, y, z right and x, y, z left, respectively), the parkinsonian subtype (TD, IP, PIGD), the medication (LEDD), the pre-operative MDS-UPDRS-III scores, age class (three classes: first age class ≤ 60; second age class > 60 ≤ 67, third age class > 67) were considered as categorical fixed-effect variables, subjects assumed as random effects and the different axial subscores as dependent variables. The approximate degrees of freedom (df) were computed according to the Satterthwaite method. Starting from an initial model containing all fixed effects, non-significant independent variables were stepwise excluded following a hierarchical backward elimination procedure based on maximum likelihood estimation (Kleinbaum et al., 2010). The final models contained the significant effects of the remaining independent variables. The generalized linear mixed models-estimated marginal means and their 95% confidence intervals (CIs) were computed for all dependent variables. As *post-hoc* tests, in case of significant fixed-effect variables, as for example DBS-lead asymmetries, their values were correlated with the post-operative changes of the different scores by non-parametric Spearman tests. As this was an exploratory pilot study, no adjustments for multiple testing were done (Bender and Lange, 2001). Adjustments for multiple comparisons are reducing type I errors at the expense of increasing type II errors. Increasing the type II errors in our study could mean that truly detrimental effects following STN-DBS treatment could be deemed as non-significant: i.e., PD-patients could truly have a poorer gait-quality due to lead asymmetry, but we ignore these finding because of multiple comparison corrections for other factors as age, pre-operative symptom severity and levodopa-responsiveness (Rothman, 1990, 2014; Perneger, 1998; Field, 2009). This would be a more problematic issue as the type I error, where no real changes of the treated patients could have been spotted just by chance (Rothman, 1990). Therefore, we report here the uncorrected results, as suggested by a number of statisticians (Rothman, 1990, 2014; Saville, 1990; Savitz and Olshan, 1995; Perneger, 1998; Bender and Lange, 2001).

## Results

### Post-operative Clinical Motor and Gait Performance

In accordance with previous studies, PD motor symptoms and LEDD were significantly reduced after STN-DBS surgery. The pre-operative LEDD (1170 ± 500 mg) decreased by 26% to 776 ± 415 mg (*p* < 0.001).

The pre-operative *MDS-UPDRS III* score was significantly impacted by DBS and medication (*F* = 62.23, *p* < 0.001). L-Dopa improved general motor symptoms pre-operatively (MED OFF 36.27 ± 14.24, MED ON 15.35 ± 9.94, *p* < 0.001) and post-operatively while active STN-DBS (STIM ON MED OFF 25.28 ± 11.92, STIM ON MED ON 17.63 ± 10.59; *p* < 0.001). STN-DBS improved general motor performance significantly by 30% compared to the pre-operative state without medication (*p* < 0.001), but not with medication. Symptom asymmetry as revealed by lateralized MDS-UPDRS items was significantly affected by L-Dopa pre-operatively (pre-op MED OFF: 0.36 ± 0.22, pre-op MED ON 0.49 ± 0.33; *p* = 0.001), but not by DBS post-operatively (post-OP MED OFF 0.36 ± 0.29). Pre-operatively, 42 PD patients (63.6%) were more severely affected on the left body side, 21 PD patients (31.8%) on the right body side, three PD patients (4.5%) revealed a symmetric motor symptom pattern, which was mostly in line with medical history of the reported subjectively perceived side of symptom onset by PD patients (87.9%). Post-operative general symptom asymmetry was not correlated with DBS lead reconstructed coordinate asymmetry in the x, y, or z-plane (*F* = 1.28, *p* = 0.289).

The *Berg balance* score was impacted by DBS and dopaminergic medication (GLM ANOVA *F* = 17.18, *p* < 0.001). There was a considerable confinement of balance pre-operatively in MED OFF (22.56 ± 4.48), which was improved during the pre-operative L-Dopa challenge (MED ON 25.95 ± 3.07, *p* < 0.001). Post-operatively, STN-DBS significantly improved balance about 6.9% to 24.36 ± 4.5 in STIM ON MED OFF (*p* = 0.009) indicating a significant improvement of balance in PD patients without medication. Further improvement of post-operative balance scores was observed with additional medication (STIM ON MED ON 25.95 ± 2.68, *p* < 0.001). However, comparison of pre- and post-operative scores in best MED ON revealed that DBS had no additional significant impact on balance performance.

Freezing of gait was significantly impacted pre- and post-operatively after STN-DBS as demonstrated by Giladi’s GFQ and rater-based FoG score. All PD patients complained about subjectively perceived freezing as assessed by the Giladi’s GFQ. The pre-operative Giladi’s GFQ score was 21.21 ± 13.57 with a reduction to 14.62 ± 13.82 post-operatively (*p* = 0.009).

Freezing of gait in PD patients was impacted by DBS and medication (*F* = 19.02, *p* < 0.001). Rater-based FoG scoring revealed pre-operative freezing phenomena in 84% of the tested PD patients, which improved significantly after suprathreshold donation of L-Dopa (MED OFF 11.84 ± 11.18, MED ON 2.94 ± 5.72, *p* < 0.001). Within the whole cohort, the degree of L-Dopa responsiveness showed remarkable variability (mean improvement 70.94 ± 65.35%) with complete resolution of FoG in 26 PD patients and worsening in two patients after L-Dopa medication. DBS improved the rater-based FoG score in MED OFF post-operatively (8.64 ± 9.68, *p* = 0.025). Interestingly, in MED ON, a significant worsening of FoG from 2.37 ± 4.23 pre-operatively to 4.67 ± 7.7 post-operatively (*p* = 0.042) was observed.

We evaluated the impact of the pre-operative LEDD, the post-operative LEDD and the relative change of LEDD after DBS on the different motor scores as MDS-UPDRS, Berg balance scale, Giladi’s GFQ and rater-based FoG score by linear regression models. There were no significant interrelations throughout all correlative LEDD and motor measures.

Thus, STN-DBS improved all motor, balance and FoG scores in MED OFF condition post-operatively, however in MED ON there was no additional benefit of STN-DBS for motor or balance improvement and even slight worsening of FoG with STN-DBS.

### The Effect of Deep Brain Stimulation Electrode Localization and Volume of Tissue Activated on Post-Operative Gait Performance

Electrode reconstructed coordinates were in the planned range with a slightly anterior position (right hemisphere: x = 12.08 ± 1.51, y = -0.5 ± 1.5, z = -1.83 ± 1.8; left hemisphere: x = 12.54 ± 1.07, y = -0.25 ± 1.57, z = -2.05 ± 1.67). The volume of tissue activated was comparable and not significantly different between left side (43.57 ± 19.45) and right side (39.82 ± 17.87, *p* = 0.22). When summing up the VTAs and correlating the sum score with the STN-DBS induced changes of the MDS-UPDRS, Berg balance score, Giladi’s GFQ score and FoG score post-operatively, there were throughout non- significant correlations indicating that the stimulation volume alone is not predictive for the post-operative outcome (all *p*-values > 0.05).

Patients were subdivided into two cohorts depending on their responsiveness to STN-DBS in terms of FoG and balance tested in the MED OFF condition. PD patients improving with STN-DBS defined by the FoG score (35 responders, pre-operative score 16.14 ± 11.73 points, post-operative score 5.66 ± 6.19; 64% improvement) revealed slightly different electrode coordinates compared to non-responders (31 non-responders, pre-operative score 6.66 ± 7.95 points, post-operative score 13.00 ± 12.09, worsening -94.71%). The electrode reconstructed coordinates on the medio-lateral x-axis (right STN 12.11 ± 1.3, left STN 12.25 ± 0.96) was slightly more medial on the left hemisphere in FoG-responders compared to non-responders (right STN 12.04 ± 1.74, left STN 12.87 ± 1.11, *F* = 5.8, *p* = 0.019). There were no differences of other electrode coordinates (y, z-axis) nor of VTAs between the two groups.

Parkinson’s disease patients improving with STN-DBS defined by the Berg balance score (42 responders, pre-operative score 21.64 ± 4.87 points, post-operative score 25.83 ± 2.0, 19% improvement) revealed no significantly different electrode reconstructed coordinates compared to non-responders (24 non-responders, pre-operative score 24.32 ± 2.97 points, post-operative score 21.35 ± 6.28; 12% worsening). Only the electrode coordinate on the medio-lateral x-axis tended to be slightly more medial in the left hemisphere (right STN 11.99 ± 1.45, left STN 12.37 ± 1.06) in Balance-responders compared to non-responders (right STN 12.23 ± 1.64, left STN 12.85 ± 1.05), however it did not reach a significant level (*F* = 3.2, *p* = 0.080). There were no differences between the other electrode coordinates (y, z-axis) nor between the VTAs of the two groups.

In summary, PD patients responding to STN-DBS in terms of FoG and balance had slightly more medially located STN electrodes.

### The Effect of Spatial Electrode Asymmetry on Post-Operative Gait Performance

To assess the impact of spatial asymmetry of the bilateral DBS electrodes on post-operative axial symptom improvement, we used a binomial distribution in the generalized linear mixed models with the fixed factors electrode asymmetry in the medio-lateral (x), anterior-posterior (y), and dorso-ventral (z) axis as well as Parkinson subtype, medication, age and pre-operative severity of the particular scale, i.e., of the MDS-UPDRS part III, of the Giladi’s GFQ score, of the FoG score and of the Berg balance score.

The post-operative change of general motor symptoms as assessed by the MDS-UPDRS was only associated with the pre-operative MDS-UPDRS score as revealed by generalized linear mixed models (Table 1). A high pre-operative MDS-UPDRS score was associated with a larger post-operative improvement (*p* < 0.001; Table 1). Neither the degree of electrode asymmetry, nor the Parkinson subtype, medication or age predicted the post-operative change.

**TABLE 1:** Fixed effects of the generalized linear mixed model for the scores of MDS-UPDRS part III, Berg balance, and freezing of gait (FoG), respectively.

Questionnaire or scale	Source	*F*	df1	df2	*p*-Values
MDS-UPDRS part III	Corrected model	21.740	1	130	<0.001
	Pre-op MDS-UPDRS score	21.740	1	130	<0.001
Berg balance	Corrected model	12.381	2	125	<0.001
	Delta x-coordinates	5.662	1	80	0.020
	Pre-op Berg balance score	24.000	1	125	<0.001
FoG	Corrected model	13.390	2	115	<0.001
	Delta x-coordinates	5.239	1	61	0.026
	Pre-op FoG score	25.065	1	125	<0.001

*The reported values for the fixed effects are degrees of freedom (df1 and df2), F and p-values.*

**FIGURE 2:**
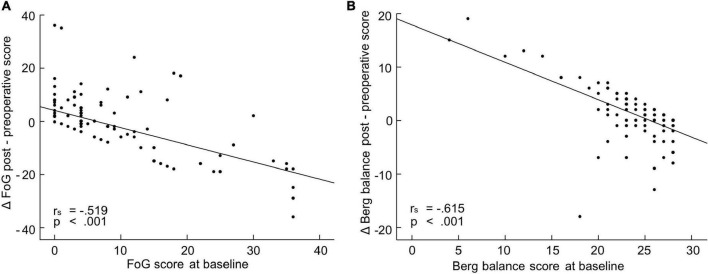
Relation of freezing of gait (FoG) and balance improvement to pre-operative symptom severity. FoG **(A)** and Berg balance **(B)** scores as recorded before and after subthalamic nucleus (STN)-deep brain stimulation (DBS) surgery. The pre-operative scores were recorded in the MED OFF condition, the post-operative scores were recorded in STIM ON/MED OFF condition. Values inset of Spearman correlation and best fit lines are given.

**FIGURE 3:**
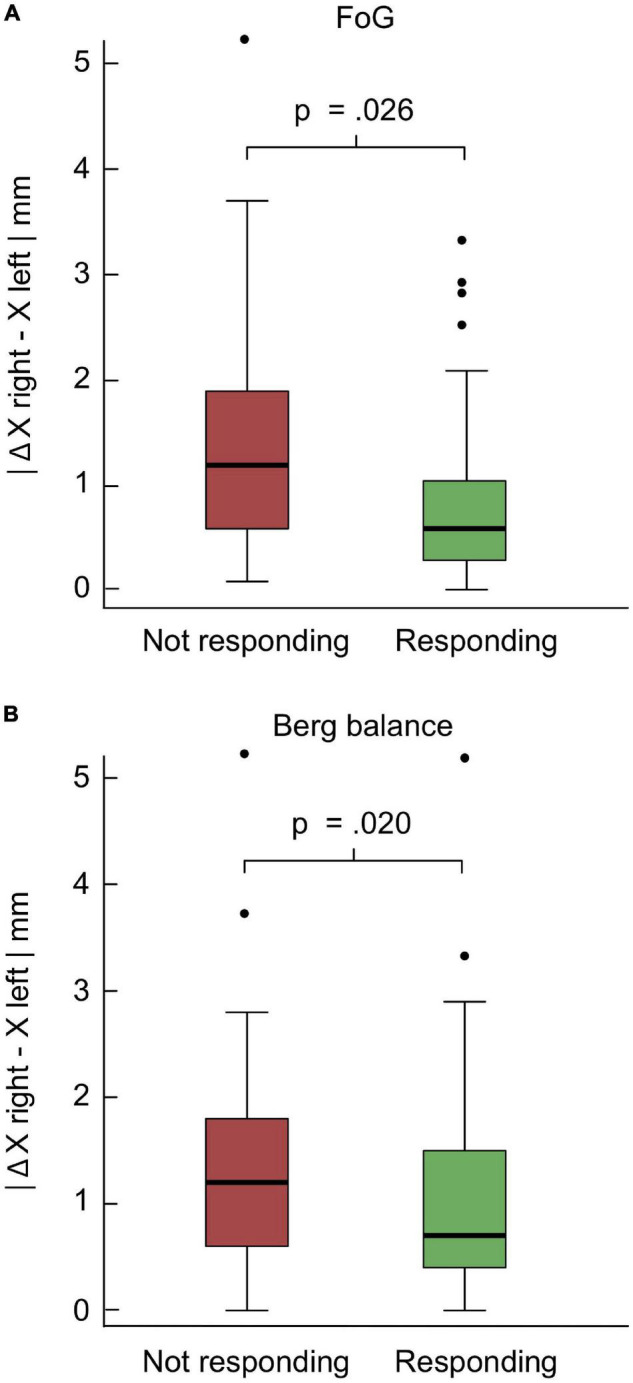
Relation of post-operative freezing of gait (FoG) and balance outcome to deep brain stimulation (DBS) electrode reconstructed coordinates. Panels **(A,B)** show the box plots of the patients subdivided into two groups depending on the responsiveness to the subthalamic nucleus (STN)-DBS treatment. If the difference between the post-operative scores minus the pre-operative scores was negative in case of FoG and positive in case of the Berg balance, indicating a post-operative improvement of the scores, the patients were assigned to the responder group, otherwise to the non-responder group. *P*-values reported in panels **(A,B)** refer to the results of the general linear mixed models.

The post-operative change of *balance* as assessed by the Berg balance score was significantly impacted by two factors, the pre-operative extent of balance disorder (*p* < 0.001; Figure 2 and Table 1) and the relative electrode asymmetry on the x axis (Table 1). Electrode asymmetry on the anterior-posterior (y) and dorso-ventral (z) axis did not affect post-operative balance outcome, however electrode asymmetry on the medio-lateral (x) axis did (*p* = 0.02; Table 1). With higher spatial asymmetry on the medio-lateral axis, there was higher probability to show no response or even a worsening of balance after STN-DBS treatment (Table 1 and Figure 3).

The same factors were predictive for the post-operative outcome of FoG measured by the rater-based FoG score. Generalized linear mixed models revealed two predictive factors which affected the post-operative change of FoG, the pre-operative severity of FoG (Figure 2) and the relative electrode asymmetry on the x axis (Table 1 and Figure 3). The higher the pre-operative FoG score, the larger was the relative post-operative change (*p* < 0.001; Table 1). Electrode asymmetry on the medio-lateral (x) axis impacted FoG improvement (*p* = 0.026; Table 1), the higher the spatial asymmetry on the medio-lateral axis, the smaller the post-operative FoG change (Figure 3). No predictive factors for the subjective Giladi’s GFQ were found.

Summarizing these findings, we found two factors predicting the responsiveness to STN-DBS in terms of balance and FoG, the pre-operative symptom severity and the extent of medio-lateral asymmetry of the electrode localization across hemispheres.

## Discussion

In this retrospective, monocentric analysis, we found STN-DBS to improve all motor, balance and FoG scores in MED OFF condition, however heterogeneous results were showed in MED ON condition. Electrode reconstructed coordinates affected the responsiveness of balance and FoG symptoms in PD patients. PD patients responding to STN-DBS in terms of FoG and balance showed slightly more medially located STN electrodes and increased medio-lateral asymmetry of the electrode coordinates across hemispheres.

There are certain limitations of the study. These were monocentric, retrospective, statistically exploratory analyses of clinical routine data of a smaller cohort of PD patients, the findings should be confirmed by a prospective, multicenter study. The electrode coordinates in x, y, z planes on CT and MR fused images were analyzed, which might be associated with methodological constraints. Due to the closely spaced anatomy of subcortical nuclei and fiber tracts, image based reconstruction method represents a rough method missing exact subcortical alignment. The asymmetry of individual brain structures across hemispheres and the post-operative shift caused by the loss of cerebrospinal fluid might hamper comparative observations of electrode positions of right and left hemispheres. We did not relate the electrode coordinates to individual fiber tracts. Current advances in neuroimaging techniques as diffusion tractography and functional connectivity enable studying normative and individual connectomes involved in the mediation of STN-DBS beneficial effects (Horn et al., 2017; Fox, 2018), since therapeutic benefit of DBS may depend on modulation of remote brain regions connected to the stimulation site (Horn et al., 2019). Recently, MR based contact lead localization and DBS programing were even optimized by machine learning algorithms depending on the characteristic brain response pattern to DBS (Boutet et al., 2021). However, these advanced neuroimaging techniques are not available at all movement disorder centers using DBS, as they are not standard procedure at our center. We focused in this study on the stereotactic routine measures of clinical procedures, which are easily available in any center. We assessed a cohort of 66 PD patients and did not find an association of DBS electrode reconstructed coordinates in x, y, z plane and general motor symptoms as reflected by MDS-UPDRS, this might be due to the small size of patients.

Further limitations might represent variability of the L-Dopa responsiveness of the PD gait disorder, different degree of L-Dopa reduction post-operatively (LEDD) and the use of acute levodopa challenges pre- and post-operatively, which might not reflect the everyday life condition with regular medication and might not rule out effects of fatigue in the non-randomized MED OFF and MED ON condition. Still, in this cohort, we did not find any impact of the LEDD or the amount of post-operative LEDD reduction on relative motor score changes, indicating that DBS is the main driver for the observed motor and gait changes. Still, in previous studies, there is an overlapping effect of dopaminergic medication and STN-DBS on the different subdomains of balance and gait adjustment (Bejjani et al., 2000; Haslinger et al., 2005; Valalik et al., 2009), so that both treatment modalities seem to restore the dysfunctional parkinsonism network with partial overlap.

The STN is subdivided into different territories as the dorsal sensorimotor area, the associative ventro-lateral area and the medio-ventral limbic part (Benarroch, 2008). Whereas dorsolateral regions of the STN receive afferent input by primary motor areas, medial subterritories are innervated by supplemental motor areas. The STN contains a segregated somatotopic body map within the sensorimotor area as revealed by intraoperative subthalamic micro-electrode recordings (Romanelli et al., 2004a). Leg-related subthalamic cells were localized in the medial STN area and tended to be situated slightly more anterior relative to arm-related cells (Romanelli et al., 2004b). This topographical organization could explain the finding of a better balance and gait response profile of medial DBS electrode reconstructed coordinates where leg-related cells are located. Besides, medial STN areas receiving SMA inputs might play an important role in the pathophysiology of the gait disorder and FoG (Bartels et al., 2006; Snijders et al., 2011).

An interesting finding was the effect of DBS interhemispheric electrode asymmetry of the right and left hemisphere on balance and gait. Although individual, anatomical, hemispherical asymmetries of the STN must be considered, one could hypothesize that different DBS electrode coordinates within the STN are associated with different drive or efficacy of divers subthalamic efferent projections resulting in asymmetric motor performance of the right and left leg. Gait asymmetry of step length or stride time is closely associated with the freezing episodes and falls (Plotnik et al., 2005; Frazzitta et al., 2013). Reduction of gait asymmetry by dopaminergic medication (Plotnik et al., 2005) or by adjustment of DBS stimulation strengths, according to the best and worst body side, improves FoG (Fasano et al., 2011). Neuronal activity of the more affected hemisphere was shown to be associated with specific cortico-subthalamic synchronization in the low-frequency band during gait with an asymmetric decoupling and breakdown during FoG in the hemisphere with less striatal dopaminergic innervation (Pozzi et al., 2019). Therefore, DBS electrode symmetry for the bilateral adequate drive of the locomotor system might be one important factor in the post-operative improvement of balance and gait.

In conclusion, post-operative outcome of PD gait characteristics after DBS is dependent on the pre-operative symptom level and electrode reconstructed coordinates, as electrode asymmetry on the medio-lateral axis.

## Data Availability Statement

The data analyzed in this study is subject to the following licenses/restrictions: monocentric clinical routine data. Requests to access these datasets should be directed to MP-N, m.poetter-nerger@uke.de.

## Ethics Statement

The studies involving human participants were reviewed and approved by Ethik-Kommission der Ärztekammer Hamburg. Written informed consent for participation was not required for this study in accordance with the national legislation and the institutional requirements.

## Author Contributions

WH and MP-N: conception of research project. FS, AG, CG, CM, MS, JK, WH, and MP-N: organization of research project. FS, AG, and MP-N: execution of research project and writing the first draft of manuscript. AG, HP, and MP-N: design, review, and critique of statistical analysis. FS, AG, HP, and MP-N: execution of statistical analysis. FS, AG, HP, CG, CM, MS, JK, WH, and MP-N: review and critique of the manuscript. All authors collaborated to carry out this work and have seen and approved the manuscript.

## Conflict of Interest

AG, CM, and WH had occasionally been reimbursed for travel expenses from Medtronic Inc. CG reported personal fees and other from Bayer Healthcare and Boehringer Ingelheim, personal fees from Abbott, Amgen, BMS, Sanofi Aventis, and Prediction Biosciences. CM received lecture, teaching, and proctoring fees from Abbott. WH received lecture fees and honoraria for serving on advisory boards and travel grants from Boston Scientific, Medtronic, and Abbott. MP-N received lecture fees from Abbott and Licher, and served as consultant for Medtronic, Boston Scientific, and Abbvie. The remaining authors declare that the research was conducted in the absence of any commercial or financial relationships that could be construed as a potential conflict of interest.

## Publisher’s Note

All claims expressed in this article are solely those of the authors and do not necessarily represent those of their affiliated organizations, or those of the publisher, the editors and the reviewers. Any product that may be evaluated in this article, or claim that may be made by its manufacturer, is not guaranteed or endorsed by the publisher.
