# Supplementary material for: Subthalamic Deep Brain Stimulation Lead Asymmetry Impacts the Parkinsonian Gait Disorder
Source: Front Hum Neurosci. 2022 Mar 28;16:788200. doi: 10.3389/fnhum.2022.788200 (PMC8995434; doi:10.3389/fnhum.2022.788200)
Supplement: Supplementary file 1 [file Table_1.pdf]

Supplementary table. Clinical and demographic characteristics of PD patients

Case Gender Age	Disease duration [years]	Pre-op LEDD [mg]	Post-op LEDD [mg]	MoCA at baseline	BDI-I at baseline	FOG pre-op med-off/ med-on	FOG post-op med-off/ med-on	BB pre-op med-off/ med-on	BB post-op med-off/ med-on	UPDRS-III pre-op med-off/ med-on	UPDRS-III post-op med-off/ med-on	Giladi pre-op	Gialdi post-op	DBS system	STN-DBS parameters<br>Left electrode<br>Right electrode	X/Y/Z coordinates<br>Left electrode<br>Right electrode
1 F 69	10	1300	511	29	1	12/0	36/36	26 /27	13 /20	28/19	38/25	NA	11	AB	3-,2.5mA,60µs,130Hz<br>3-,1.7mA,60µs,130Hz	13.9/-0.9/-1.3<br>-12.7/-0.8/-3.9
2 M 72	15	NA	899	28	13	9/0	7/0	24/20	28/27	33/16	50/29	40	16	AB	2-,1.7V,60µs,130Hz<br>2-,1.6V,60µs,130Hz	12.9/-1.1/-1.3<br>-9.6/-0.6/-4.4
3 M 72	13	666	NA	28	3	8/4	20/18	26/28	26/28	29/18	23/21	9	8	AB	2-,2.7mA,60µs,130Hz<br>2-,2.8mA,60µs,130Hz	14/-0.4/-1.9<br>-12/0.8/-3.6
4 M 70	5	1690	1630	20	NA	8/4	6/6	24/27	24/28	26/18	32/28	15	23	AB	4-,2mA,60µs,130Hz<br>3-,2V,60µs,130Hz	14.3/-0.8/-1.3<br>-14.4/-1.3/-1.1
5 F 60	9	160	400	30	NA	9/0	6/8	24/25	27/25	32/15	34/25	NA	8	AB	2-,2.5mA,60µs,130Hz<br>3-,3.2mA,60µs,130Hz	11.5/2.2/-3.2<br>-11.8/-1.3/-3.1
6 M 57	5	200	0	NA	11	6/0	NA/0	26/28	NA/28	33/18	20/5	3	1	BS	2-,4.8mA,60µs,130Hz<br>2-,1.6mA,60µs,130Hz	12.3/4.1/-2.8<br>-11.2/1.3/-4.8
7 F 73	15	1345	440	29	28	13/4	24/4	21/23	23/28	35/17	26/6	10	35	BS	2-,3.6mA,50µs,130Hz<br>2-,3-,2.5mA,60µs,130Hz	12.2/-0.5/-1.9<br>-9.7/0.4/-3.5
8 F 56	10	699	300	26	7	0/0	NA/0	26/28	NA/28	34/16	NA/9	16	6	BS	3-,4mA,60µs,130Hz<br>3-,2.4mA,60µs,130Hz	13.5/-2.2/-1.2<br>-11.6/-0.1/-0.2
9 M 52	12	1513	580	30	2	6/0	0/NA	26/28	27/NA	20/3	19/NA	10	NA	BS	3-,3.4mA,60µs,119Hz<br>3-,3.8mA,60µs,119Hz	11.2/1.3/-2.9<br>-11.1/1.9/-3
10 M 65	15	1042	732	26	8	35/2	17/10	14/25	26/27	48/17	13/10	NA	30	BS	1-,2.4mA,60µs,130Hz<br>2-,2.7mA,60µs,130Hz	12.1/0.9/-3.5<br>-11.6/0.2/-1.6
11 F 64	15	1292	316	NA	NA	NA/NA	5/7	NA/NA	24/24	72/59	28/27	NA	6	BS	1-,2mA,60µs,170Hz<br>1-,2.7mA,60µs,130Hz	12.9/0.1/-4.4<br>-10.9/1.6/-3.3
12 M 56	7	662	470	26	NA	0/0	0/0	28/28	28/28	21/19	16/9	NA	2	BS	2-,7mA,60µs,176Hz<br>2-,2.5mA,60µs,176Hz	13.3/-2.3/-2.5<br>-9.6/0.5/-3.6
13 M 49	11	1847	1231	28	28	17/0	25/6	26/27	17/25	37/9	38/17	NA	29	BS	1-,2.5mA,60µs,95Hz<br>1-,2.5mA,60µs,95Hz	1.9/0.7/-2.8<br>-12.4/0.3/-3.4
14 M 66	9	732	632	30	14	8/4	0/0	26/28	28/28	32/19	15/12	11	1	BS	3-,3.5mA,60µs,130Hz<br>3-,2.7mA,60µs,130Hz	12.3/0/-2.9<br>-13.2/-0.4/-2.2
15 M 61	8	750	1625	30	NA	36/16	11/0	4/10	19/22	53/23	27/26	NA	28	BS	3-,2.3mA,60µs,169HZ<br>2-,2.8mA,60µs,169Hz	12.1/-1/-0.7<br>-11.5/-2.3/-0.6

16 F 62	9	1315	1483	NA	10	6/2	6/4	21/24	24/26	24/6	15/6	13	18	BS	3-,3mA,60µs,174Hz<br>3-,3.4mA,60µs,174Hz	11.6/-1.7/-1.3<br>-8.7/-1.4/-1.8
17 M 66	15	610	457	28	NA	7/1	0/0	26/28	28/28	38/19	28/28	17	0	BS	2-,2.2mA,60µs,130Hz<br>2-,2.2mA,60µs,130Hz	13/0.3/-3.5<br>-10.7/1.2/-4
18 M 63	18	1408	305	27	8	7/4	0/0	24/24	27/24	15/12	6/14	NA	12	BS	2-,2.2mA,60µs,130Hz<br>2-,2.1mA,60µs,130Hz	11.3/0.2/-4.7<br>-11.9/0.9/-4.5
19 F 66	8	110	837	27	6	12/11	6/6	24/28	28/28	30/16	17/15	19	22	BS	2-,2.6V,60µs,112Hz<br>2-,2.8V,60µs,112Hz	12.9/0.4/-2.4<br>-12.9/0.1/-2.4
20 F 68	6	455	800	26	11	14/0	11/16	23/27	23/27	19/13	28/21	26	34	BS	2-,3.2mA,60µs,95Hz<br>2-,3.4mA,60µs,95Hz	11.4/0.7/-4<br>-10.8/-0.4/-4.2
21 M 67	11	1739	1810	29	4	19/11	36/20	18/20	0/13	32/12	64/44	35	NA	BS	4-,3.1mA,60µs,130Hz<br>4-,3.2mA,60µs,130Hz	11.3/-1.4/-0.9<br>-12.9/-2.5/1.1
22 M 51	9	466	830	30	12	27/4	18/8	21/24	24/27	64/24	32/33	40	NA	BS	2-,3.5mA,60µs,130Hz<br>2-,3.8mA,60µs,130Hz	10.9/1.5/-4<br>-8/1.3/-3.8
23 M 66	16	850	1064	26	7	7/0	14/7	25/26	26/27	30/16	36/17	24	23	BS	3-,2.6mA,60µs,130Hz<br>3-,3.1mA,60µs,130Hz	14.1/0.5/1<br>-13/1.7/0.2
24 M 58	13	400	400	19	17	0/0	13/10	23/27	27/27	39/26	23/18	NA	27	BS	2-,1.9V,60µs,130Hz<br>2-,1.3mA,60µs,130Hz	11.8/-0.8/-1.8<br>-10.4/0.8/-1
25 M 65	9	986	405	27	3	0/0	0/0	25/28	26/26	17/4	25/8	4	NA	ME	1-,2.8V,60µs,130Hz<br>1-,2.9V,60µs,130HZ	12.5/0.3/-3.5<br>-12/1.2/-2.7
26 F 59	8	1120	250	25	8	25/32	12/NA	22/24	23/NA	38/27	30/NA	29	31	ME	1-,3.2V,60µs,70Hz<br>1-,3.2V,60µs,70Hz	12.2/0.4/-3.5<br>-12/0.7/-3.5
27 F 70	9	1622	1131	NA	14	8/0	0/0	23/28	20/22	29/12	23/14	8	NA	ME	2-,2V,60µs,125Hz<br>3-,0.5V,60µs,125Hz	13/-1.1/-2.2<br>-11.2/-1.3/-2.1
28 M 54	10	1120	575	NA	10	4/2	6/0	21/26	28/28	42/12	8/4	16	1	ME	2-,3.8V,60µs,130Hz<br>2-,2V,60µs,130Hz	13.5/-0.7/-1.9<br>-13.3/-1.5/-3.3
29 M 71	7	1110	650	28	7	4/18	9/0	21/18	17/26	55/40	30/10	38	NA	ME	3-,1.8mA,60µs,130Hz<br>3-,1.8mA,60µs,130Hz	11.9/0.7/-4.2<br>-12.6/-1.2/-3.5
30 M 73	8	907	433	NA	3	10/1	4/0	25/27	27/28	30/8	17/12	14	4	ME	1-,2.5V,60µs,130Hz<br>1-,2.9V,60µs,130Hz	11.7/0.5/-2.3<br>-11.5/0.9/-2.4
31 F 49	13	1633	325	NA	21	22/15	6/5	20/26	22/22	71/32	29/21	49	43	ME	1-,1.7mA,60µs,125Hz<br>2-,3.5mA,60µs,125Hz	9.8/0.1/-3<br>-12.6/0.4/-2.1
32 M 74	13	1110	566	NA	9	25/1	6/0	21/25	27/27	41/19	24/22	46	3	ME	1-,2.7V,60µs,130Hz<br>1-,2.3V,60µs,130Hz	11.9/0.2/-4.4<br>-12.6/-1.1/-2.4
33 M 64	14	1036	600	23	9	4/0	0/0	23/28	27/27	19/4	5/3	12	15	ME	3-,2mA,60µs,125Hz<br>3-,1.5mA,60µs,125Hz	12.8/-2.7/-1.1<br>-12.4/-3.7/-2.6
34 F 66	11	714	651	22	16	1/0	6/2	22/25	27/27	21/9	21/16	20	5	ME	2-,2.7V,60µs,130Hz<br>1-,2.7V,60µs,130Hz	11.4/-0.2/-0.1<br>-10.6/-1.4/-1.2

35 M 69	9	1845	1420	NA	18	17/0	0/0	24/28	24/27	52/19	27/11	40	NA	ME	1-,2.7V,60µs,125Hz,<br>1-,2.8V,60µs,125Hz	12.4/0.1/-2.8<br>-11.3/-0.7/-2.4
36 M 72	6	1300	500	26	NA	8/3	6/12	24/28	23/20	33/13	45/42	NA	15	ME	2-,3V,60µs,130Hz<br>3-,2V,60µs,130Hz	12/-0.5/-1.8<br>-14.5/0/1.6
37 M 54	12	1498	681	20	9	3/0	9/2	26/28	27/28	35/7	17/1	8	3	ME	3-,2.9V,90µs,130Hz<br>2-,2.9V,60µs,130Hz	11.6/-3/0.5<br>-10.4/-1.3/-3
38 F 65	8	1208	842	NA	11	36/0	18/0	20/26	25/26	48/6	30/16	36	11	ME	1-,3.1V,60µs,130Hz<br>3-,3.4V,60µs,130Hz	11.1/-1.7/-2<br>-13/-3.1/-0.5
39 M 54	8	2415	1155	27	11	5/4	4/0	24/28	26/28	55/28	17/6	35	13	ME	2-,2.7mA,60µs,130Hz<br>2-,3.3mA,60µs,130Hz	13.5/-3.5/-1<br>-12.8/-1.9/-1.6
40 M 54	10	NA	842	27	2	2/0	0/0	25/28	27/28	26/2	27/19	2	3	ME	1-,2.9V,60µs,130Hz<br>1-,2.9V,60µs,130Hz	12.2/0.1/-1.5<br>-11.5/-1.2/-1.6
41 F 65	10	920	820	26	3	30/14	32/NA	17/25	NA/NA	53/11	50/NA	41	NA	ME	2-,2.3V,60µs,130Hz<br>2-,1.8mA,60µs,130Hz	14.8/1.4/-3.4<br>-12.8/-1.8/-1.7
42 F 62	17	1324	859	28	10	0/0	0/0	23/28	26/26	34/11	37/20	NA	26	AB	2-3-,3.7mA,60µs,130Hz<br>3-4-,2mA,60µs,130Hz	13.8/-2.2/2.4<br>-9.3/1.6/-1.7
43 F 63	12	900	998	NA	16	0/0	2/0	26/28	27/26	24/6	24/18	16	21	AB	1-2-,3.3mA,60µs,150Hz<br>1-2-,3.3mA,60µs,130Hz	10.7/-0.1/-3.1<br>-11.1/0.4/-1.2
44 F 69	15	2462	1042	26	19	24/0	5/6	16/25	24/24	30/8	13/10	NA	13	AB	2-3-,2.5mA,60µs,130Hz<br>2-3-,2.5mA,60µs,130Hz	14/-1.6/-1.7<br>-12.5/0.4/-1.8
45 M 59	14	1350	931	28	15	15/0	5/0	22/28	27/27	36/3	19/22	33	10	BS	2-4-,2.2V,60µs,170Hz<br>2-,2.1mA,60µs,170Hz	11.5/1.8/-3.1<br>-12.1/1.6/-3.8
46 F 76	11	1150	210	30	18	13/0	3/0	21/21	22/24	38/26	20/8	19	9	BS	2-3-,3.5mA,60µs,170Hz<br>2-3-,3mA,60µs,170Hz	13.1/0.4/-0.6<br>-13.7/-2.1/0
47 M 56	6	800	660	28	13	3/1	0/0	25/27	28/28	30/6	12/4	15	NA	BS	1-2-,5.9mA,60µs,130Hz<br>1-2-,4.9mA,60µs,130Hz	12.4/1.1/-3<br>-12/0.5/-2.7
48 M 54	9	1197	840	NA	NA	36/4	0/2	6/28	25/28	64/16	17/7	NA	7	BS	3-,3mA,60µs,130Hz<br>2-3-,3.6mA,60µs,130Hz	10.7/0.8/-7.1<br>-9.7/3.4/-5.1
49 F 66	9	1354	449	30	10	0/0	6/0	28/28	27/28	14/6	19/8	11	3	BS	1-3-,2.9mA,60µs,130Hz<br>1-3-,3.1mA,60µs,130Hz	11.9/0.7/-3.5<br>-11/-1.3/-2.5
50 M 63	7	944	299	30	11	3/0	14/6	22/24	23/27	30/13	29/21	NA	19	BS	3-,3mA,60µs,179Hz<br>2-3-,4mA,60µs,179Hz	15.1/1.5/-0.4<br>-15.2/-0.4/1.2
51 F 65	7	1319	849	25	NA	0/0	0/0	27/28	27/28	9/2	7/2	5	3	BS	3-,2mA,60µs,154Hz<br>3-4-,4.6mA,60µs,154Hz	13.5/-2/-0.8<br>-14.1/-3.5/1
52 F 56	17	810	405	28	9	33/12	18/8	28/27	26/27	31/14	27/15	48	10	BS	2-3-,2.7mA,60µs,130Hz<br>3-,3.4mA,60µs,130Hz	11.2/0.2/-1<br>-11.8/0.3/-0.4
53 M 49	9	1039	1039	28	NA	7/0	0/0	25/28	27/28	53/22	33/29	NA	NA	ME	1-3-,1.5V,60µs,120Hz<br>2-3-,1.5V,60µs,120Hz	13.6/-2.3/-0.3<br>-15.1/-2.6/2.5

54 M 65	10	1687	1432	30	NA	36/0	7/10	12/26	25/27	63/8	20/17	0	8	ME	0-1-,3.6V,60µs,130Hz	11.9/1/-2.3
															0-1-,2.9V,60µs,130Hz	-11.8/0.9/-2.1
55 M 72	19	1305	1365	30	17	7/0	13/4	26/27	23/23	28/16	14/17	29	25	ME	0-,2.5V,60µs,130Hz	12.4/-1.3/-3
															0-,2V,60µs,130Hz	-10.8/0.1/-3.3
56 M 55	10	2670	1510	NA	3	35/4	19/14	23/28	26/26	55/17	39/35	51	NA	ME	0-1-,2.5V,60µs,60Hz	12.6/0.9/-3.4
															2-,2.7V,60µs,60Hz	-12.8/-2.2/1.5
57 F 70	12	1092	985	23	3	4/0	0/0	22/28	21/24	26/7	37/25	5	NA	ME	1-2-,2.5V,60µs,130Hz	12.4/1.1/-1.7
															1-2-,2.5V,60µs,130Hz	-11/1.5/-1.1
58 F 69	7	433	630	30	10	4/0	3/NA	23/27	25/NA	28/6	19/NA	9	NA	ME	1-2-,1.2V,60µs,180Hz	11.5/0.6/-3.2
															1-2-,2.3V,60µs,180Hz	-12.5/0.2/-2.3
59 F 50	6	1075	650	29	21	0/0	0/0	26/27	27/27	32/14	19/6	NA	10	ME	1-2-,2.6V,60µs,130Hz	12.8/0.2/-2.8
															1-2-,2.5V,90µs,130Hz	-11.4/-0.2/-0.7
60 M 68	7	873	616	NA	NA	9/2	12/4	21/27	27/26	26/8	33/34	NA	NA	ME	1-2-,2V,60µs,210Hz	12.6/0.1/-2.8
															3-,3.8V,90µs,210Hz	-13/-1.1/-3.1
61 M 67	8	1312	949	28	8	22/0	0/8	23/23	NA/16	32/14	NA/8	28	32	ME	1-2-,1.2V,60µs,130Hz	12.8/0.2/-2.7
															1-2-,1.2V,60µs,130Hz	-11.4/1/-5.4
62 M 70	7	850	1260	24	5	18/1	36/36	23/25	22/23	33/15	31/37	45	56	ME	1-,3.2V,60µs,70Hz	14.7/1.7/-5.2
															1-2-,3V,60µs,70Hz	-16.7/2/-0.1
63 M 61	19	1862	1463	NA	NA	NA/NA	30/18	NA/NA	20/27	56/37	59/35	NA	NA	ME	0-3-,2V,60µs,130Hz	12.7/0.4/-0.6
															0-2-,2.1V,60µs,130Hz	-11.1/0.3/-1.4
64 M 69	6	NA	614	NA	4	4/3	7/0	26/28	22/26	38/8	14/5	11	3	ME	2-3-,2.2V,60µs,160Hz	13.9/-1.1/0.2
															2-3-,2.6V,60µs,160Hz	-14.6/-0.9/-0.8
65 M 66	11	1042	300	26	6	0/4	3/2	19/22	25/26	63/33	33/28	6	6	ME	1-2-,3.6V,60µs,125Hz	11.8/1.2/-3.9
															3-,0.8V,60µs,125Hz	-13.9/1-.3/-0.1
66 M 62	3	1596	765	28	24	15/0	0/0	22/27	27/28	32/14	7/4	NA	NA	ME	1-2-,2.8V,60µs,130Hz	12.6/0.3/-1.4
															1-,1.7V,60µs,130Hz	-10.8/0.1/-3.3

In "Disease duration [years]" column, the disease duration is calculated from the date of diagnosis to the date of baseline measurement. In “STN-DBS parameters” column, values reported are active contacts, amplitude (V = volts or mA = milliampere), pulse width (µs = microseconds) and stimulation frequency (Hz), for the left and right electrode. The neurostimulator case was set as positive (anode) and the active contacts as negative (cathodes, contact number followed by a minus sign). For the left and right Medtronic (ME) electrodes, contact 0 was the most ventral and contact 3 was the most dorsal. For the left and right Boston Scientific (BS) and Abbot (AB) electrodes, contact 1 was the most ventral and contact 4 was the most dorsal. In “X/Y/Z coordinates” column, electrode coordinates are given in relation to the AC-PC line (mm) lateral to the midline (X), posterior to the mid-commissural point (Y) and inferior to the inter-commissural plane (Z). Abbreviations: BDI- I: Becks Depression Inventory; Berg balance: Berg Balance scale score; FOG: Freezing of Gait Assessment Course score; H&Y: Hoehn & Yahr scale; LEDD: levodopa equivalent daily dose, conversion factors used for the calculation of LEDD from Tomlinson et al. (2010); MoCA: Montreal Cognitive Assessment score; NA: not available; UPDRS-III: motor-subscore (part III) of the Unified Parkinson’s Disease Rating Scale of the Movement Disorder Society.
